# Differential expression of p120-catenin 1 and 3 isoforms in epithelial tissues

**DOI:** 10.1038/s41598-018-36889-w

**Published:** 2019-01-14

**Authors:** Jan-Hendrik Venhuizen, Sebastian Sommer, Paul N. Span, Peter Friedl, Mirjam M. Zegers

**Affiliations:** 1 Department of Cell Biology, RIMLS, Radboud university medical center, Nijmegen, The Netherlands; 2 Radiotherapy & OncoImmunology laboratory, department of Radiation Oncology, RIMLS, Radboud university medical center, Nijmegen, The Netherlands; 3 Cancer Genomic Centre, University Medical Center Utrecht, Utrecht, The Netherlands; 4 David H. Koch Center for Applied Research of Genitourinary Cancers, The University of Texas MD Anderson Cancer Center, Houston, Texas USA

## Abstract

P120 catenin (p120) is a non-redundant master regulatory protein of cadherin-based cell-cell junctions, intracellular signaling, and tissue homeostasis and repair. Alternative splicing can generate p120 isoforms 1 and 3 (p120-1 and p120-3), which are implicated in non-overlapping functions by differential expression regulation and unique interactions in different cell types, with often predominant expression of p120-1 in mesenchymal cells, and p120-3 generally prevalent in epithelial cells. However, the lack of specific p120-3 protein detection has precluded analysis of their relative abundance in tissues. Here, we have developed a p120-3 isoform-specific antibody and analyzed the p120-3 localization relative to p120-1 in human tissues. p120-3 but not p120-1 is highly expressed in cell-cell junctions of simple gastrointestinal epithelia such as colon and stomach, and the acini of salivary glands and the pancreas. Conversely, the basal layer of the epidermis and hair follicles expressed p120-1 with reduced p120-3, whereas most other epithelia co-expressed p120-3 and p120-1, including bronchial epithelia and mammary luminal epithelial cells. These data provide an inventory of tissue-specific p120 isoform expression and suggest a link between p120 isoform expression and epithelial differentiation.

## Introduction

Cadherins are transmembrane cell-cell adhesion receptors with crucial roles in development, morphogenesis, tissue homeostasis and cancer. A key regulator of cadherin function is p120 catenin (p120), an armadillo (ARM)-related protein, which controls the retention and stability of classical cadherins at the plasma membrane by binding directly to the cytoplasmic tail of cadherins^1^. Besides cell-cell interactions, p120 regulates gene transcription through several transcription factors including Kaiso^2^, and the activity of Rho family GTPases and downstream cytoskeletal dynamics. In mice, germline deletion of p120 is embryonic lethal^3,4^, suggesting critical and non-redundant functions, and conditional deletion of p120 using the Cre/LoxP system causes early lethality or dramatic developmental defects in epithelial and endothelial tissues such as the vasculature^5^, colon and intestine^6^, salivary gland^7^, mammary gland^8,9^ and the proximal tubules of the kidney^10^. Deletion of p120 further leads to inflammation in the skin, the intestinal tract, pancreas and lung^6,11–15^ as well as tumor initiation or progression in epithelial organs, including salivary gland^7^, skin^11,12^, esophagus^14^, mammary gland^9^ and liver^16^. The severity and variability of these outcomes is tissue-dependent and may depend on the level and type of cadherins expressed and co-expressed p120 family members, such as ARVCF, δ-catenin, p0071, and plakophilins 1–3^3^.

An additional important but poorly understood level of regulation of p120-dependent cell- and tissue functions depends on differential expression of p120 isoforms generated from its single *CTNND1* gene. Alternative splicing-dependent use of four different translation initiation sites results in p120 isoforms 1, 2, 3 and 4 (p120-1-4)^17,18^. The longest isoform p120-1 contains a 101 amino acid N-terminal domain including a coiled-coil motif which is lacking in p120-3. Since p120-1 and p120-3 have differential affinities for binding partners, including transcription factors such as Kaiso and DIPA^2,19^ and regulators of Rho GTPase signaling^20^, their differential expression patterns likely reflect functional differences, including opposing effects of p120 isoforms on proliferation and cell migration^21–24^. Therefore, a detailed analysis of p120 isoform expression *in situ* in human tissues is of interest. Previous analyses by Western blot^18^, whole-tissue RT-PCR analysis^17^ and, indirectly, by immunofluorescence in tissues using p120-1-specific and pan-p120 antibodies^25^, have indicated that most cells express multiple p120 isoforms, with p120-3 often prevalent in differentiated epithelial cells, in combination with variable expression of p120-1. However, the direct detection of p120-3 localization in tissues at the cellular level is precluded by the lack of a p120-3-specific antibody. As technical challenge, compared to p120-1, p120-3 lacks a unique amino acid sequence, and this has complicated the development of selective antibodies.

Here, we describe the development of polyclonal p120-3-specific antibodies using a strategy that uses the free N-terminal amino acid as a unique epitope of p120-3. Using this antibody together with a commercial p120-1-specific monoclonal antibody, we directly compare expression and localization of p120 isoforms in human epithelial and non-epithelial tissues by immunofluorescence. We show that whereas p120-3 expression is generally associated with E-cadherin expression, several E-cadherin-positive epithelial cells express p120-1 as the dominant form, particularly epithelial compartments with a lower differentiation state or high turnover, such as the basal layer of the skin and bronchial epithelia. In addition, differential p120-1 and p120-3 expression is detected in distal and proximal tubuli of the kidney, and p120-3, but not p120-1 is absent from non-epithelial tissues. These data for the first time directly compare p120 isoform expression in human tissues, and suggest a link between p120 isoform expression and differentiation state.

## Materials and Methods

### Immunogen design

**Figure 1:**
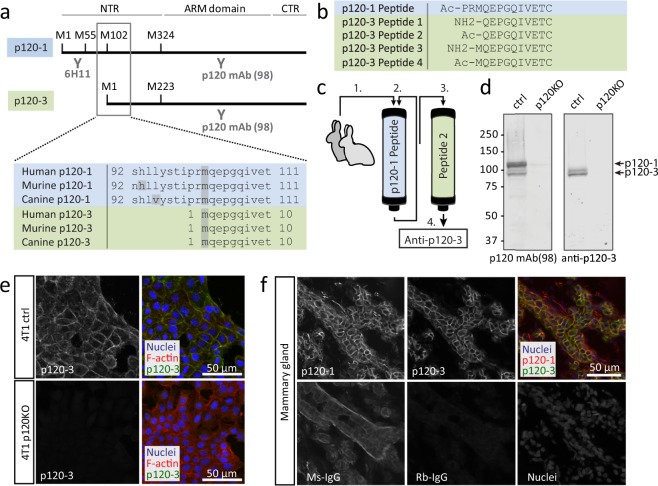
Generation and characterization of p120-3-selective pAb: strategy and specificity testing. (**a**) Domain structure and antibody recognition sites of p120 isoforms. p120 consists of an N-terminal region (NTR), an Armadillo (ARM) domain and a C-terminal region (CTR). Alternative entry points (corresponding to methionines at amino acid positions 1, 55, 102 and 324 for p120-1) lead to protein isoforms differing in the NTR. Established monoclonal antibodies target the N-terminus of p120-1 and p120-2 (6H11) or the ARM domain (p120 mAb (98)). Alignment of human, murine and canine sequences from the NCBI database show that the p120-3 N-terminus, starting with the methionine (m) indicated in grey shading, is highly conserved. (**b**) In green, peptides designed to represent different potential p120-3 N-termini, modified by acetylation and/or methionine cleavage are indicated (p120-3 peptide 1–4). The blue-shaded top row shows the p120-1 peptide, which includes the two amino acids (PR) directly preceding the p120-3 initiator methionine. (**c**) After immunization with the peptide 1–4 mix, the sera of the two rabbits were pooled and depleted for anti-p120-1 antibodies by passing them several times over the p120-1 column (blue). Upon p120-1 depletion, anti-p120-3 was purified by affinity purification using immobilized p120-3 peptide 2 (green). (**d**) Western blot analysis of 4T1 cells expressing endogenous p120 (ctrl) and 4T1 p120 knock-out cells (p120KO). The Western blots were incubated with either p120 mAb (98), which recognizes all p120 isoforms, or anti-p120-3 (indicated below each blot). Full length blots are shown in Supplementary Fig. 13. (**e**) Immunofluorescence of 4T1 ctrl and p120KO with anti-p120-3. (**f**) Immunofluorescence of an FFPE section of a human mammary gland using 6H11 and anti-p120-3. An adjacent section was incubated with species-matched non-targeting control IgGs (Ms-IgG and Rb-IgG).

A distinct chemical difference between p120-3 and p120-1 is the presence of a free amino acid at the N-terminus of p120-3 whereas a peptide bond is present at the corresponding amino acid in p120-1 (Fig. 1A). We therefore used the N-terminus of p120-3 as a unique epitope for specific antibody development. The first ten amino acids corresponding to the human p120-3 N-terminus show 100% homology to the N-terminal amino acid sequence of p120-3 in mouse and dog (UniGene, NCBI), and were selected as antigen (Fig. 1A). Cleavage of the initiator methionine and/or N-terminal acetylation are processes that may occur in over 50% of eukaryotic proteins^26^, but it is unknown whether the p120-3 N-terminus is subject to these modifications. We therefore combined four different peptides representing the four possible N-termini (peptides 1–4, Fig. 1B) of p120-3, for immunization.

### Generation and purification of p120-3-specific antibodies

Peptide synthesis, coupling to Keyhole Limpet Hemocyanin (KLH), immunization, serum collection and automated affinity chromatography were performed by ImmunoGlobe GmbH, Germany. Briefly, peptides representing p120-3 N-termini (Fig. 1A,B) were synthesized with purity >98%, except for p120-3 peptide 1. The free N-terminal glutamyl residue of this peptide was subject to pyroglutamyl formation, as occurs *in vivo*^27^, resulting in a purity of 76.8% with a 22.5% contribution of the pyroglutamyl form. The identity of the peptides was confirmed by mass spectrometry. An equimolar mix of p120-3 peptides 1–4 was conjugated to KLH via an extra C-terminal cysteine residue. Two female rabbits aged six and nine months of a proprietary strain were immunized intradermally with the KLH conjugate in PBS containing on average 117 μg of each of the four peptides, emulsified 1:1 with complete Freund’s adjuvant. The rabbits were boosted with half that dose, each, after 2, 4, 8, 10, 12, 28, 31, and 35 weeks, with Montanide ISA 206 as an adjuvant and in week 33 with antigen emulsified in Montanide ISA 50V2. Test sera were taken after week 6, aimed at characterizing antibodies targeting the different potential N-termini. Regular sera were collected 14, 16, 18, 37, 39, and 41 weeks after the first immunization.

For affinity purification, individual peptides were conjugated via the C-terminal cysteine residue to Sulfolink Iodoacetyl resin (ThermoFischer). For initial proof-of-concept experiments, test sera taken at week 6 after initial immunization were affinity purified on a tandem array of five columns, with the p120-1 peptide matrix comprising an immobilized peptide corresponding to amino acids P100 - T111 of the p120-1 sequence, followed by matrices with p120-3 peptides 1 to 4 in the indicated order (Supplementary Fig. 1). Total yields of the different affinity-purified fractions showed that in either rabbit over 80% of antibody was recovered from the p120-1 peptide column (Rb1-p120, Rb2-p120), indicating that the majority of the raised anti-p120 antibodies did not specifically recognize the free N-terminus of p120-3 (data not shown).

To prepare the final anti-p120-3 antibodies, which was used for all stainings, regular sera were pooled, diluted in PBS, and antibodies cross-reacting with p120-1 were depleted by multiple adsorption/elution cycles on the p120-1 peptide matrix until no protein in the eluate was detected. Next, p120-3-specific antibodies were affinity purified on a resin with p120-3 peptide 2, the eluate was dialyzed to PBS, and passed again over the p120-1 peptide matrix for additional depletion of potentially remaining p120-1 cross-reacting antibodies. The resulting antibody was quantified photometrically (1.4 OD^280^ ≙ 1.0 mg/ml) and concentrated by ultrafiltration, stabilized with 1 mg/ml BSA and 0.02% NaN_3_, and sterile filtered.

### Antibodies and fluorescent dyes

In addition to the p120-3 isoform-specific rabbit polyclonal antibodies described in this work, antibodies against p120 included mouse monoclonal p120, clone 98 (p120 mAb (98), BD Biosciences cat no. 610134), which recognizes all p120 isoforms, and 6H11 (Santa Cruz sc-23873), a mouse monoclonal antibody against p120-1 and p120-2^28^, which in most tissues recognizes a single isoform corresponding with p120-1 on Western blot (WB)^25^. Rabbit polyclonal p120-3 antibodies and the Rb1- and Rb2-p120 were diluted to 1 µg/ml for WB, 1.5 µg/ml for immunofluorescence (IF) of cells and 5 µg/ml for IF of tissues, p120 mAb (98) was used 0.25 µg/ml for WB and 0.5 µg/ml for IF and 6H11 was used 4 µg/ml for IF. Additional antibodies used in this study are: CD34 (Abcam Ab8536, 1.33 µg/ml for IF), Vimentin (Abcam 24525, diluted 1:400 for IF), wide spectrum Cytokeratin (PCK, Abcam Ab9377, diluted 1:200 for IF), E-cadherin (BD Biosciences 610182, 2.5 µg/ml in IF), and E7 mouse monoclonal anti-β-tubulin (Developmental Studies Hybridoma Bank, University of Iowa, USA) was used as a loading control for WB. Secondary antibodies for Odyssey detection comprised Alexa Fluor 680-conjugated or Alexa Fluor IRDye 800-conjugated goat anti-mouse and goat anti-rabbit IgG (Invitrogen). To visualize nuclei and F-actin, 4′,6-Diamidine-2′-phenylindole dihydrochloride (DAPI, Roche 10236276001, 5 µg/ml) and Alexa Fluor™ 488-conjugated Phalloidin (ThermoFischer, 1:200) were used, respectively. Secondary antibodies for immunolabeling of cells and tissues comprised Alexa Fluor™ 546 or 647 goat anti-mouse IgG (H + L), Alexa Fluor™ 488, 546 or 647 goat anti-rabbit IgG (H + L), Alexa Fluor™ 488 goat anti-chicken IgY (H + L) and Alexa Fluor™ 555 donkey anti-rabbit IgG (H + L) conjugates (Invitrogen).

### Cell culture

MDCK parental, p120 knock-down and murine p120 re-expression cell lines were a generous gift from A. Reynolds^29^, and were grown in MEM (Gibco) supplemented with 10% FCS, 10.000 U/ml penicillin/streptomycin and 2 mM L-glutamine at 37 °C, 5% CO_2_. NMuMG cells were grown in DMEM, supplemented with 10% FCS, 10.000 U/ml penicillin/streptomycin, 2 mM L-glutamine and 10 µg/ml insulin at 37 °C, 10% CO_2_. 4T1 cells were grown in RPMI 1640 (Gibco) supplemented with 10% FCS, 10.000 U/ml penicillin/streptomycin and 1 mM sodium pyruvate at 37 °C, 5% CO_2_.

### Generation of 4T1 p120 KO cells by CRISPR/Cas9

4T1 cells were transfected using Lipofectamine 2000 (Invitrogen) 24 hours after plating with 2 µg of a pool of three p120 CRISPR Cas9 KO constructs. Each of these constructs encodes a Cas9 nuclease and GFP driven by a chicken β-actin hybrid promoter, and one of the following gRNAs under the control of a U6 promoter, and derived from the GeCKO v2 library: gRNA1 TCTGGTCCGATTGCTCCGAA, gRNA2 TGTGGTCTCCGTGCGTCTAG, gRNA3 TGATGGGACCACTAGACGCA targeting three different sites in the *Ctnnd1* gene (Santa Cruz sc-419478). As a control, cells were transfected with a non-targeting scrambled gRNA construct (Santa Cruz sc-418922). Media were changed 6 h after transfection, and after growth for an additional 72 h in antibiotics-free medium, single GFP-expressing cells were collected in a 96 wells plate using a DB Aria flow cytometry sorter and grown for 3 weeks until colonies appeared. P120 KO colonies were selected after 3 weeks based on Western blot analysis.

### Western blot analysis

Cells grown for 48 h to 95% confluence in 6 well plates were washed once with PBS prior to lysis. Subsequently, cells were scraped in Laemlli sample buffer (100 mM Tris-HCl, 4% SDS, 20% glycerol, 200 mM DTT, bromophenol blue) and proteins were denatured by heating at 95 °C for 5 minutes.

Proteins were separated by SDS-PAGE and transferred to PVDF membrane. Membranes were blocked with 5% BSA in PBS with 0.2% Tween-20 (PBST) and incubated overnight at 4 °C with primary antibodies in 5% BSA in 0.2% PBST. The membranes were subsequently washed six times with 0.2% PBST and incubated with Alexa Fluor-conjugated secondary antibodies (Thermo Fischer) for 1 h at room temperature. The blots were washed six times with 0.2% PBST and once in PBS. The blots were scanned using the Odyssey CLx imaging system (Li-Cor, Lincoln, NE) and analyzed with the Image Studio Lite software Ver 4.0. Uncropped blots are shown in Supplementary Fig. 13.

### Immunofluorescence of cultured cells

Cells grown for 48 h on coverslips were fixed for 15 min at 37 °C with 4% paraformaldehyde in 0.1 M phosphate buffer pH 7.4. After blocking with 10% normal goat serum and 0.3% Triton X-100, cells were incubated with primary antibody for 2 h at room temperature, and subsequently with Alexa Fluor-conjugated secondary antibodies (Thermo Fischer), DAPI and Alexa Fluor™-conjugated phalloidin (ThermoFischer) to visualize nuclei and F-actin, for 1 h at room temperature. Coverslips were mounted on object slides with FluorSave (Calbiochem), and cells were imaged with a Leica DMI6000B using a HC PL APO 63x oil objective and DFC365FX camera. Confocal imaging was performed with a Fluoview FV1000 microscope (Olympus) using a LUMPLFL 40x water objective.

### Immunoprecipitation of p120 isoforms

Cells grown for 72 h to 100% confluence in 10 cm dishes were washed twice with ice-cold PBS prior to lysis on ice with 1 ml IP lysis buffer (125 mM NaCl, 20 mM Hepes pH 7.4, 1% Nonidet P40 substitute, 5 µg/ml pepstatin, 10 µg/ml chymostatin, 3 µg/ml leupeptin, 10 µg/ml antipain, 0.5 mM benzamadin, 0.2 mM PMSF, 0.1 kU/ml aprotinin, 1 mM Na_3_VO_4_, 1 mM NaF). Insoluble debris was removed by centrifugation for 15 min at 15,000 g, 4 °C. Lysates were precleared with CL-4B beads (GE Healthcare, Piscataway, NJ) for 30 min at 4 °C. As total lysate control, 5% of the cleared lysate was used. The remaining supernatant was incubated with 1-2 µg of antibody for 1 h at 4 °C. Sepharose-Protein G was added and the lysates were incubated overnight at 4 °C while rotating. The beads were washed five times with lysis buffer by centrifugation. Proteins were eluted from the beads by heating for 5 minutes in sample buffer with 100 mM DTT at 95 °C and the samples were, together with the lysates (5% of total), analyzed by SDS-PAA gel electrophoresis and Western blotting

### Human tissues

4 µm tissue sections of human kidney were provided by the department of Pathology, Radboud university medical center Nijmegen. These samples comprised anonymised left-over tissue from routine treatment at our Academic Hospital, used with permission of the IRB of the Radboudumc according to national law and the Code of Conduct of the Federation of Medical Scientific Societies in the Netherlands (Code for Proper Secondary Use of Human Tissue in the Netherlands). Only tissues from patients that did not object to the use of their biomaterials for academic research were used. Tissue samples from the human mammary gland comprised normal tissue of breast cancer patients adjacent to tumor tissue. These tissues were obtained, with informed consent, from the Radboud Breast Cancer Biobank, approval 2013/576 IRB (Institutional review board) Radboud university medical center. All other tissue samples were from a commercial human tissue microarray containing 23 × 2 mm cores (Bio SB; bsb0298).

### Haematoxylin and eosin (H&E) staining of formalin-fixed tissue

Formalin-fixed paraffin-embedded (FFPE) tissue sections were deparaffinized by incubating sequentially three times for 5 min in xylene, and subsequent incubations in 100%, 96%, 70% and 50% ethanol and distilled water, each twice for 1 min. Next, the sections were incubated with haematoxylin for 20 min. The samples were washed for 15 min in running water, and subsequently incubated 5 min with eosin. The samples were washed briefly in water, and dehydrated by sequentially incubating twice for 1 min in 50%, 70%, 96% and 100% ethanol, and three times 1 min in xylene. The sections were imaged using the Pannoramic Flash 250III (3D Histech), using a Plan-Apochromat 20x objective and a CIS VCC-FC60FR19CL camera.

### Immunofluorescence of formalin-fixed tissue

4 µm thick FFPE tissue sections were deparaffinized by incubating sequentially three times for 5 min in xylene, twice 5 min in 100% and 96% ethanol, 1 min in 70% and 50% ethanol, and 10 min in flowing distilled water. Subsequently, antigen retrieval was performed by incubating 15 min in Tris-EDTA buffer (10 mM Tris, pH 9.0, 1 mM EDTA) heated to 95 °C. Samples were allowed to cool for 1 h at room temperature, washed with PBS for 1 h, and blocked with 10% normal goat serum, 1% BSA and 0.2% Triton X-100 in PBS. The sections were incubated with primary antibody overnight at 4 °C in 1% BSA/ 0.05% PBST, and with Alexa Fluor-conjugated secondary antibodies and DAPI in the same buffer for 1 h at room temperature. As a negative control, an adjacent tissue section was incubated with species-matched non-targeting (NT) primary IgGs. Antibody incubation steps were followed by washing three times 5 min with 0.05% PBST. Coverslips were mounted onto the sections with Fluoromount-G (ThermoFischer). The sections were imaged using the Pannoramic Flash 250III (3D Histech), using a Plan-Apochromat 20x objective and a pco.edge 5.5 4MP camera, the DMI6000B (Leica) using an HCX PL S-APO 20.0 × 0.50 DRY objective or an HCX PL S-APO 40.0 × 0.75 DRY objective with a DFC360FX camera, or the Fluoview FV1000 (Olympus) with a UPLSAPO 60x oil objective.

## Results

### Validation of the anti-p120-3 purification protocol

To validate our approach towards purification of p120-3 antibodies two rabbits were immunized with a mix of four peptides representing differentially modified p120-3 N-termini (Fig. 1A,B). An aliquot of serum of either rabbit was depleted of antibodies recognizing p120-1, and p120-3-recognizing antibodies were subsequently isolated from the p120-1-depleted antiserum by sequential affinity purification using immobilized p120-3 peptides 1–4 (Supplementary Fig. 1). The performance and specificity towards p120-3 of the resulting eight antibody fractions as analyzed by Western blot, immunoprecipitation and immunofluorescence was variable (Supplementary Fig. 1 and data not shown). As unexpected result, we found that all antibody fractions recognized p120-3 in an assay in which we immunoprecipitated p120-3 with one antibody followed by detection with each other antibody fraction (data not shown), or with a anti-p120 monoclonal recognizing all isoforms (Supplementary Fig. 1) indicating that the different N-termini of p120-3 species were not recognized differentially.

### Purification and characterization of anti-p120-3 antibody

To improve the yield of anti-p120-3 antibody with highest possible specificity, we selected immobilized peptide 2 as the single affinity column to purify-p120-3 from p120-1-depleted serum, based on a strong detection of p120-3 and a lack of cross-reactivity with p120-1 in all three techniques used in either rabbit. To further minimize cross-reactivity to p120-1 and increase recognition of p120-3, additional immunizations were administered to the rabbits. Next, antibodies recognizing p120-1 were depleted from pooled rabbit sera by multiple depletion rounds with a p120-1 peptide column, followed by affinity purification using immobilized peptide 2 (Fig. 1C). The resulting antibody fraction (“anti-p120-3”) was used in all subsequent experiments.

Anti-p120-3 specifically detected p120-3 by Western blotting as shown using lysates of wild-type 4T1 murine mammary cancer cells and 4T1 p120 KO cells generated by CRISPR/Cas9-mediated knock-out of *Ctnnd1* (Fig. 1D). Immunoblotting of anti-p120-3 immunoprecipitates showed detection by all previously generated fractions from the peptide 1–4 affinity columns (Supplementary Fig. 2), indicating that anti-p120-3 recognizes the total cellular pool of p120-3. Immunofluorescence labeling of 4T1 control and 4T1 KO cells also demonstrated specific staining (Fig. 1E). To determine its suitability for application in FFPE human tissue, anti-p120-3 was applied in routine histological samples and compared to binding of mouse anti-p120-1 monoclonal antibody 6H11^28^. Both antibodies strongly labeled the membrane of mammary epithelial cells, compared to diffuse low-level background signal obtained by species-matched non-targeting IgG (Fig. 1F). A strong signal for anti-p120-1, but not anti-p120-3, was observed in capillaries, larger blood vessels and the glomerulus of the kidney, and occasionally in stromal cells (Supplementary Figs 3 and 4), consistent with previous reports^25^. Thus, anti-p120-3 antibody recognizes murine and human p120-3, does not cross-react with p120-1, and can be applied in analyses by Western blot, immunoprecipitation and immunofluorescence in cells and human FFPE tissue.

### P120-1 and p120-3 expression in normal human tissues

#### Co-expression of p120-1 and p120-3 in ciliated pseudostratified epithelia but not in gastrointestinal simple epithelia

**Figure 2:**
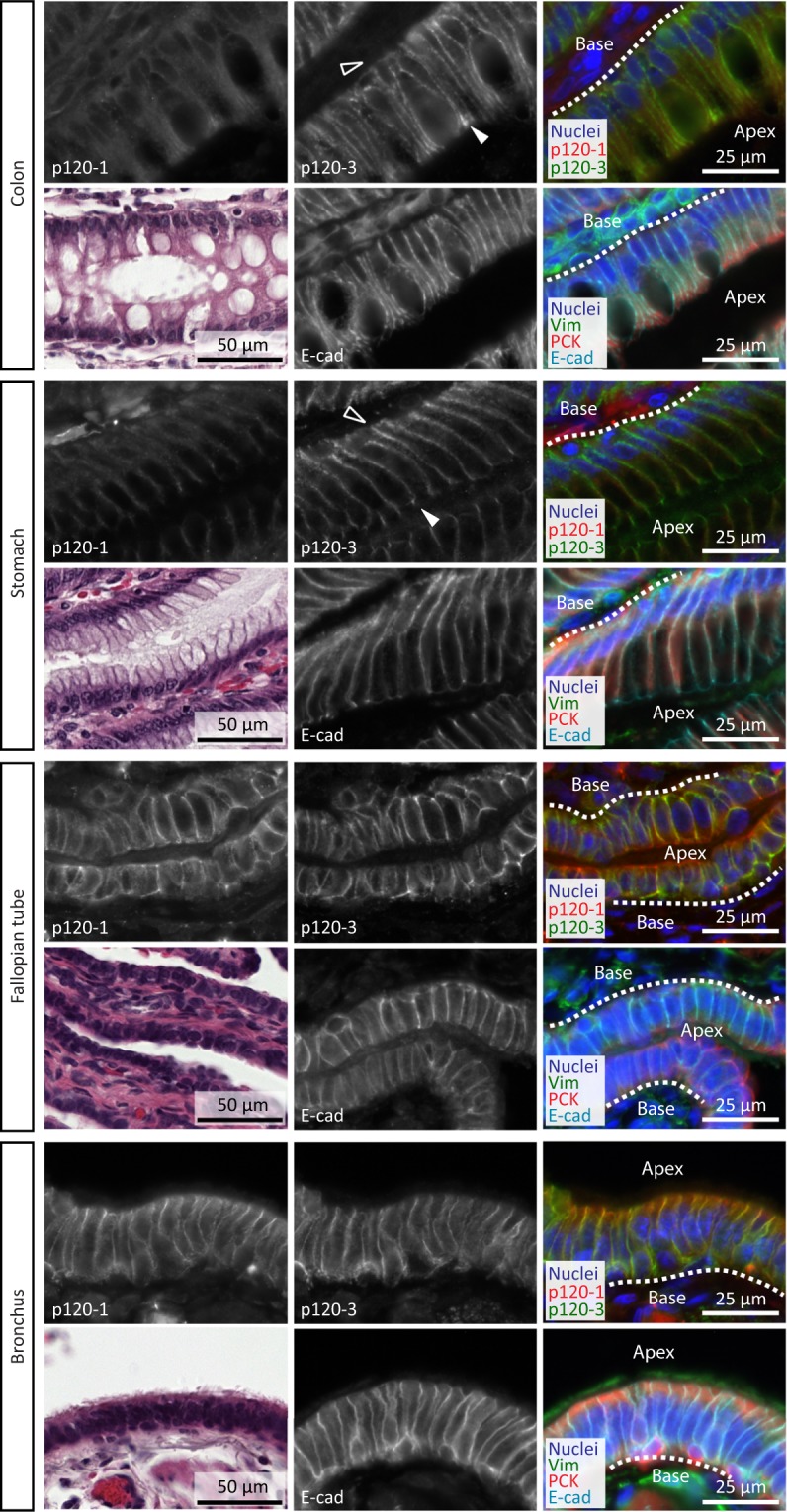
p120 isoform localization in simple and pseudostratified epithelia. Panels show p120-1 (6H11 mAb) and p120-3 (anti-p120-3 pAb) expression in the colon, stomach, fallopian tube and bronchus, and PCK, E-cadherin and H&E in adjacent sections. Open and filled arrowheads indicate apical (filled, apex) and basal (open, base) subregions of the epithelial cell-cell adhesion.

Gastrointestinal simple epithelia of the stomach and colon were identified to predominantly express p120-3, which showed a slightly increased intensity along the plasma membrane at the base and apex, in colocalization with E-cadherin (Fig. 2, arrowheads). Staining of p120-1 was below the detection limit in these epithelia but blood vessels from the same sections were positive for p120-1 (Supplementary Fig. 5), indicating that low p120-1 staining was not due to technical issues. In contrast to colon and stomach, p120-1 strongly stained the pseudostratified ciliated epithelia of the fallopian tube and bronchus, which co-expressed p120-3 and p120-1 along the entire lateral surface from apex to base (Fig. 2). Thus, in these examples of simple and pseudostratified epithelia we found consistent co-expression of E-cadherin and p120-3, whereas levels of p120-1 are variable. The differential detection of both isoforms in different tissues further emphasizes the selectivity of anti-p120-3 antibodies for analysis of archival FFPE material.

#### p120-1 is co-expressed with p120-3 in ducts but not acini of glandular epithelia

**Figure 3:**
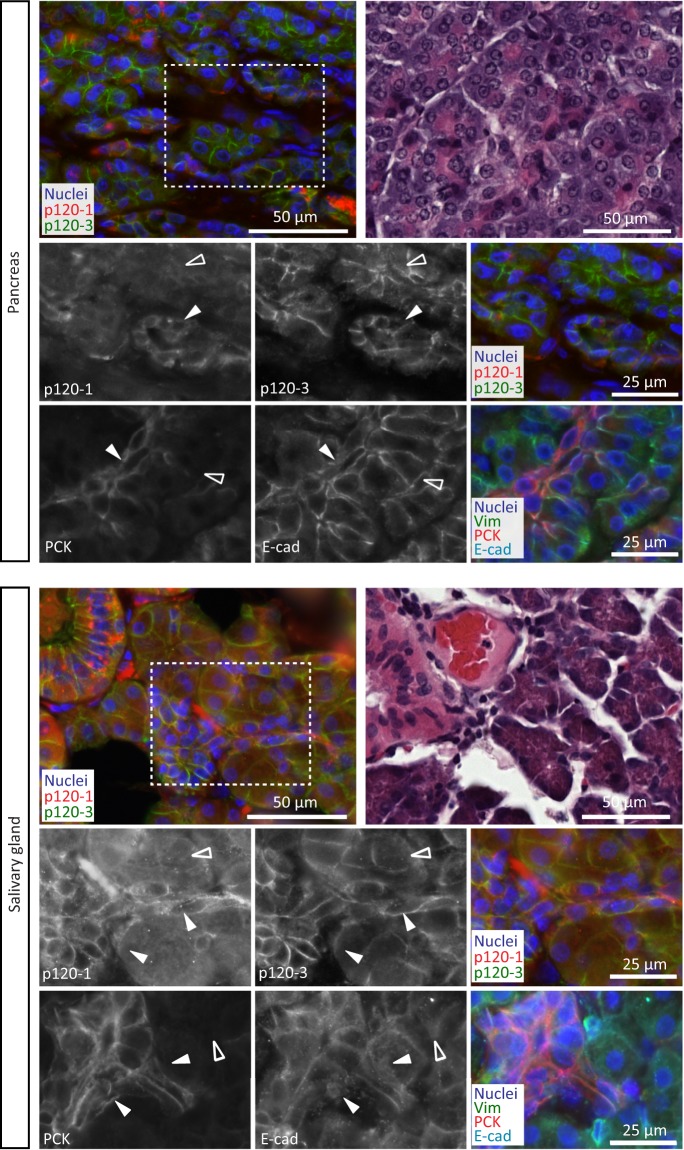
p120 isoform localization in glandular tissue. Panels show p120-1 (6H11 mAb) and p120-3 (anti-p120-3 pAb) expression in the pancreas and a salivary gland, and vimentin, PCK and E-cadherin and H&E in adjacent sections. Open and filled arrowheads indicate ducts (filled) and glandular acini (open). Lower panels are magnifications of the area indicated by the dashed boxes.

Differential expression of p120 isoforms was further detected in the ducts and acini of glandular tissue of the pancreas, parotid salivary gland and prostate gland (Fig. 3, Supplementary Fig. 6). Acinar epithelial cells of these tissues expressed p120-3, with low or no p120-1 and pan-cytokeratin (PCK) expression as detected by a broad spectrum antibody that reacts with a variety of epithelial cell types (Fig. 3, open arrowheads). By contrast, the ductal compartment of the pancreas and salivary gland co-expressed p120-3 and p120-1 (Fig. 3, closed arrowheads; Supplementary Fig. 6). Both glandular and ductal epithelia expressed E-cadherin expression at similar levels.

The glandular tissue derived from ectodermal appendages, including sweat glands and the mammary gland^30^, co-expressed both p120-1 and p120-3, in addition to E-cadherin and PCK (Supplementary Fig. 7). At subcellular resolution, p120-1 and p120-3 localized to both lateral and basal plasma membrane of mammary epithelial cells (Supplementary Fig. 7), indicating coexistence of both p120 isoforms along both adhesions between luminal cells and adhesions between luminal cells and the myoepithelial layer.

#### Differential expression of p120-1 and p120-3 in kidney tubules

**Figure 4:**
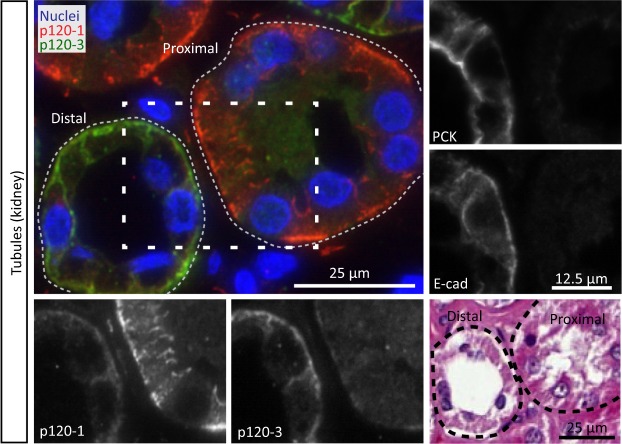
p120 isoform localization in the proximal and distal tubules of the kidney. Panels show p120-1 (6H11 mAb) and p120-3 (anti-p120-3 pAb)expression in a proximal tubule and a distal tubule, and PCK, E-cadherin and H&E in adjacent sections.

The different segments of the kidney tubules differentially express p120 isoforms. Distal tubules predominantly expressed p120-3, with low or no p120-1, whereas proximal tubules expressed p120-1, with very low p120-3 (Fig. 4). N- and E-cadherin expression was reported to segregate to proximal and distal tubules of the human kidney, respectively^31^ and the different p120 isoform expression may reflect differential engagement in epithelial- or mesenchymal-type adherens junctions^18^. Consistently, E-cadherin and PCK were expressed in distal but not in proximal tubules (Fig. 4).Taken the results in simple and pseudostratified epithelia together, although p120-3 often associates with E-cadherin expression, the link between p120-1  and E-cadherin is more variable.

#### Differential expression of p120-1 and p120-3 within stratified epithelia

**Figure 5:**
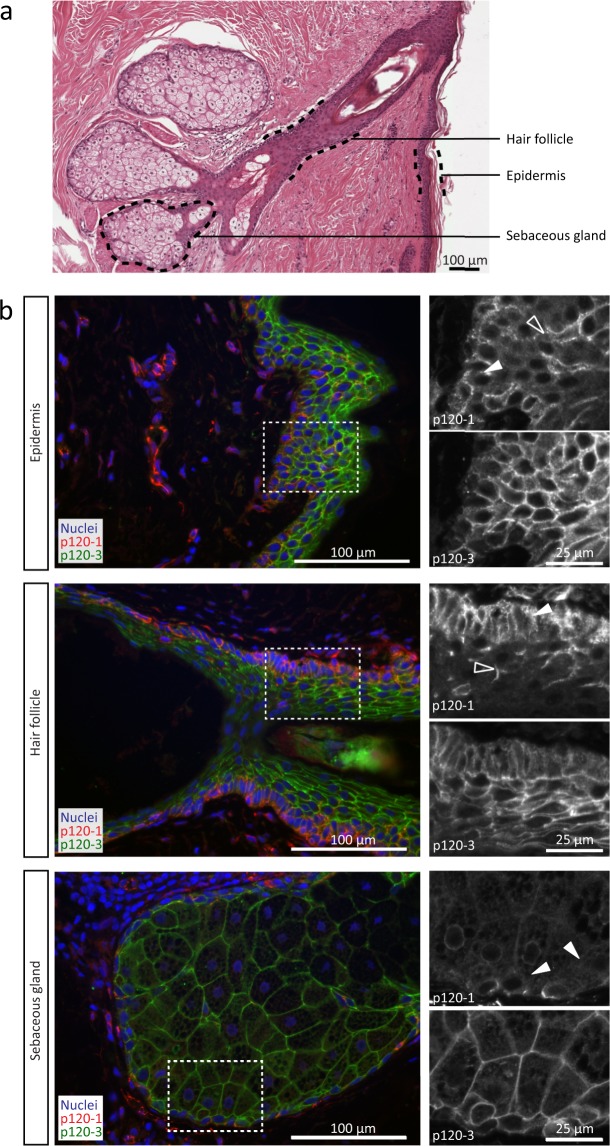
p120 isoform localization in the skin. (**a**) Histological section of epidermis, containing a hair follicle and sebaceous glands. (**b**) p120-1 (6H11 mAb) and p120-3 expression (anti-p120-3 pAb) in the epidermis, the root sheath of a hair follicle and a sebaceous gland. Panels on the right represent a magnification of the area in the dashed boxes. Open and filled arrowheads indicate p120-1-positive cells in the basal layer (filled) and intermediate layers (open).

p120-3 was strongly expressed in all epithelial cells of the epidermis, hair follicles and sebaceous glands, together with E-cadherin and PCK, but intensity was reduced in the basal layer (Fig. 5, Supplementary Fig. 8). p120-1 showed comparably inverse expression, with peak intensity in basal compartments and no signal in upper epithelial layers. Occasional vimentin- and p120-1-positive cells were observed within the upper layers, likely representing intraepidermal Langerhans cells^32^ (Fig. 5, Supplementary Fig. 8). The p120-1-positive basal epithelial cells, including the basal layer of the epidermis, the outer root sheath of the hair follicle and cells covering the sebaceous gland contain multipotent precursor cells^33^, thus suggesting a switch from p120-1 to p120-3 expression with epidermal differentiation. Differential p120-1 and p120-3 expression was further detected in basal and stratified cells of tonsil but not cervix epithelium (Supplementary Fig. 9). This indicates that p120-3 is largely absent in the stem-cell-containing basal layer but is upregulated with stratification of multilayered epithelia^33^.

#### p120-3 expression in the liver is restricted to the bile duct

In the liver, co-expression of p120-3, E-cadherin and PCK was restricted to the cholangiocytes of the bile ducts, which further lacked p120-1 (Supplementary Fig. 10). By contrast, both p120-3 and p120-1 were largely absent in hepatocytes, despite strong expression of E-cadherin (Supplementary Fig. 10).

#### p120-3 expression in other tissues

The densely T-cell-populated tissues of the thymus and tonsil showed sporadic punctate p120-1 distribution, and were negative for p120-3 and E-cadherin (Supplementary Fig. 11). Notably, p120-1 was enriched in the germinal centre, in a pattern that is reminiscent of (epithelio)reticulocytes^34^. E-cadherin and p120-3, but not p120-1, were further expressed in the junctions between the syncytiotrophoblasts that form a syncytium at the outer layer of the chorionic villus and cytotrophoblasts of the placenta (Supplementary Fig. 12). In the seminiferous tubule p120-1 was expressed in the vimentin-positive outer layer of spermatogonia, but gradually reduced towards the centre and completely absent in differentiated spermatids (Supplementary Fig. 12). P120-3 and E-cadherin were neither expressed in the testis nor neuronal tissue of the cerebellum (data not shown). Thus, p120-3 is mostly absent from non-epithelial tissues.

## Discussion

Here we show that isoform-specific antibodies can be raised to p120-3 based on its N-terminus, despite its otherwise identical amino acid sequence to p120-1. The direct co-labeling of both p120 isoforms provides complementary insights into p120 isoform expression, whereas previous studies relied on an indirect subtractive approach cross-referencing anti-p120-1 labeling against pan-p120 detection^25,32^.

We considered four different p120-3 N-termini that may arise due to cleavage of the initiator methionine, and/or N-terminal acetylation^26^. All four antibody fractions recognized cell-derived p120-3, which suggests that the p120-3 antibody fractions raised against different N-terminal peptides are polyspecific. Specificity towards p120-3 and not p120-1 may be due to sterical inability to accept the bulky charged arginine residue, located immediately N-terminally of the initiator methionine of p120-3 in the p120-1 polypeptide in the binding pocket^35^.

By immunolocalization analyses of both p120-1 and p120-3 in a sampling of mostly epithelial human tissues we confirm previously observed variance of p120-3 and p120-1 co-expression in different epithelia, as demonstrated by protein or mRNA expression analyses or inferred by immunofluorescence^17,25,36,37^. Specifically, we show highly prevalent p120-3 expression in terminally differentiated cells including excretory glandular cells and non-basal epidermal cells, and in the stomach and colon in conjunction with E-cadherin-positive adherens junctions, the latter of which is in accordance with previous immunolabelings of other gastrointestinal epithelia^25^. Preferential expression of p120-1 or co-expression of p120-3 and p120-1 is demonstrated in basal layers of epithelia of the skin and skin appendages including the hair follicle, sebaceous gland, sweat gland and mammary gland^30^, as well as in pseudostratified epithelia with high turnover, such as bronchial epithelium.

The relevance of varying co-expression of p120 isoforms is currently unclear. Differentiation and tissue homeostasis in many of the epithelia in which we find relative high p120-1 expression is controlled by canonical Wnt signaling including the skin, proximal kidney tubule, and fallopian tube^38–41^. P120 is structurally related to β-catenin, a key regulator of the Wnt pathway^3^ and p120-1, but not p120-3, contains a Wnt signaling destruction sequence causing p120-1 stabilization in the presence of Wnt signaling^42^. Nuclear signaling by p120 isoforms has been implicated in canonical Wnt signaling by their ability to regulate Kaiso-mediated gene expression and controlling the expression of, and response to, Wnt ligands in conjunction with β-catenin^43,44^. These findings suggest highly complex p120 isoform-dependent regulation of Wnt signaling. Future work will clarify whether high p120-1 expression is maintained through Wnt signaling in these tissues and the functional correlations between differential p120 isoform expression, Wnt signaling regulation and tissue homeostasis.

Application of direct p120-1 and p120-3 detection will be useful in understanding the tissue-specific functions of p120 isoforms. Tissue-specific isoform expression patterns, and associated differential regulation of isoform-specific function likely underlie the highly variable phenotypes obtained in conditional knock-out studies in mice. While some mouse knock-out phenotypes, such as loss of epithelial integrity and resulting inflammatory responses likely depend on shared functions of p120 isoforms, in particular stabilization of cadherins^5,6^, other phenotypes may be due to isoform-specific functions. For example, ablation of p120 in the salivary gland^7^ or pancreas^15^ results in reduced acinar differentiation and increased ductal content. The differential isoform expression in glandular ducts and acini may affect differentiation, for instance via Wnt signaling as discussed above. Similarly, the p120 knock-out-induced phenotype observed in proximal, but not distal tubules^10^, may be related to the different isoforms expressed in this tubular segments. Isoform-specific functions may also explain tissue-dependent susceptibility to oncogenic transformation upon p120 knock-out, as seen in salivary gland^7^, skin^11,12^ and the mammary gland^9^, which may be due to differences in nuclear signaling or cytoskeletal regulation.

The regulation of p120 isoform expression, for instance through ESRP1 and 2^45^, and the functional significance of p120-3 and p120-1 expression in development and cancer warrants further work. Isoform-specific knock-out models based on removal of specific exons^46^ or targeted mutation of specific initiator methionines, may elucidate the extent of compensation and exclusive functions in an *in vivo* context.

## Supplementary information

Supplementary Figures

## Data Availability

The datasets generated and/or analyzed during the current study are available from the corresponding author on reasonable request.
