# Supplementary material for: Differential expression of p120-catenin 1 and 3 isoforms in epithelial tissues
Source: Sci Rep. 2019 Jan 14;9:90. doi: 10.1038/s41598-018-36889-w (PMC6331582; doi:10.1038/s41598-018-36889-w)
Supplement: Supplementary file 1 — Supplementary Figures [file 41598_2018_36889_MOESM1_ESM.pdf]

Differential expression of p120-catenin 1 and 3 isoforms in epithelial tissues

Jan-Hendrik Venhuizen[1], Sebastian Sommer[1], Paul N. Span[2], Peter Friedl[1,3,4], Mirjam M. Zegers[1,]*

[1] Department of Cell Biology, RIMLS, Radboud university medical center, Nijmegen, the Netherlands;

[2] Radiotherapy & OncoImmunology laboratory, department of Radiation Oncology, RIMLS, Radboud university medical center, Nijmegen, The Netherlands;

[3]Cancer Genomic Centre, University Medical Center Utrecht, Utrecht, The Netherlands;

[4]David H. Koch Center for Applied Research of Genitourinary Cancers, The University of Texas MD Anderson Cancer Center, Houston, Texas, USA;

*Correspondence: mirjam.zegers@radboudumc.nl

Supplementary information

Supplementary Figure 1. Specificity testing of different affinity-purified p120-3 antibody fractions. a) Overview of the purification of p120-3-specific antibodies. Two rabbits were immunized with a mixture of peptides 1-4. An aliquot of serum of either rabbit was depleted of p120-1 using immobilized p120-1 peptide. Eluates from the p120-1 column were named Rb1-p120 and Rb2-p120. Anti-p120-3 antibodies of the p120-1-depleted sera were purified by successive affinity purification on immobilized peptides 1, 2 3 and 4. Antibody eluates are named after rabbit (Rb1 or Rb2) and immobilized peptide (P1,P2,P3,P4). b) Western blot analysis of MDCK parental cells (par), MDCK cells stably expressing shRNA against p120 (p120kd) and p120kd cells overexpressing murine p120-1 (mp120-1) or p120-3 (mp120-3). Blots were stained with a commercial mouse monoclonal antibody (clone 98 anti-p120; p120 mAb(98)), Rb1-p120 or Rb2-p120. Note that p120 mAb Rb1-p120 and Rb2-p120 recognize all p120 isoforms. Arrows indicate p120-1 and p120-3. β-tubulin was used as loading control. c) Lysates of mp120-1 and mp120-3 were probed with the different antibody eluates from the immobilized p120-3 peptide columns. d) Immunoprecipitation (IP) of p120-3, using the antibody fractions indicated above the lane and detected using p120 mAb (98). Uncropped Western blots are shown in Supplementary Fig. 13.

Supplementary Figure 2. Immunoprecipitation with p120-3 antibody. Cell lysates of 4T1 cells were used to immunoprecipitate p120-3 using anti-p120-3 antibody. Western blot analysis show detection by previously generated P1-P4 antibody fractions from rabbit 2 serum.

Supplementary Figure 3. High expression of p120-1 in endothelial cells. a) Adjacent sections of a blood-vessel rich area of human mammary gland showing co-staining for p120-1 (6H11 mAb) and p120-3 (anti-p120-3) or with H&E. Inserts represent an enlargement of the dashed boxes. b) p120-1 positive blood-vessels co-express the endothelial marker CD34.

Supplementary Figure 4. p120 isoform expression in the glomerulus. a) Histological section of the kidney, stained with H&E, showing a glomerulus. b) Immunofluorescence labeling of the glomerulus. Panels show staining for p120-1 (6H11 mAb) and p120-3 (anti-p120-3), and vimentin and E-cadherin from an adjacent section.

Supplementary Figure 5. p120 isoform expression in colon and stomach. Images show a larger area of tissue as represented in Figure 2. Histological sections from the colon and stomach were labeled for p120-1 (6H11 mAb) and p120-3 (anti-p120-3). Note that epithelial cells of stomach and colon express very low levels of p120-1, whereas nearby blood vessels (arrowheads) are strongly positive for p120-1.

Supplementary Figure 6. p120 isoform localization in a pancreatic duct and an acinus of the prostate gland. Panels show p120-1 (6H11 mAb) and p120-3 (anti-p120-3), and vimentin, PCK and E-cadherin and H&E from adjacent sections. Filled and open arrowheads indicate ducts (filled) and glandular acini (open).

Supplementary Figure 7. p120 isoform localization in the mammary gland and sweat gland. a) p120-1 (6H11 mAb) and p120-3 (anti-p120-3) localization in a mammary duct and lobular units. Lower panels show PCK and E-cadherin from an adjacent section. b) High-resolution confocal microscopy of lobular epithelial cells. Cell-cell contacts are present containing both p120-1 and p120-3 (closed arrowhead) or only p120-1 (open arrowhead). As the latter is formed between peripheral cells with elongated nuclei, it may represent a homotypic myoepithelial contact. c) P120 isoform localization in the sweat gland. Panels show p120-1 (6H11 mAb) and p120-3 (anti-p120-3), and vimentin, PCK and E-cadherin and H&E in adjacent sections.

Supplementary Figure 8. Localization of vimentin, PCK and E-cadherin in the epidermis, hair follicle and sebaceous gland. Filled and open arrowheads indicate vimentin-positive cells in the basal layer (filled) and intermediate layers (open), which possibly represent melanocytes and Langerhans cells, respectively [32]. Arrow indicates PCK surrounding a lipid-filled vacuole.

Supplementary Figure 9. p120 isoform localization in the stratified epithelia of the cervix and tonsil. Panels show p120-1 (6H11 mAb) and p120-3 (anti-p120-3), and vimentin, PCK and E-cadherin and H&E in adjacent sections. Filled and open arrowheads indicate p120-1-positive cells in the basal layer (filled) and intermediate layers (open).

Supplementary Figure 10. p120 isoform localization in the liver. a) H&E staining of a section of liver, containing a portal vein, bile ducts and hepatocytes. b) Panels show (6H11 mAb) and p120-3 (anti-p120-3) expression in a bile duct and hepatocytes, and vimentin, PCK and E-cadherin in an adjacent section.

Supplementary figure 11. p120 isoform localization in the tonsil. a) H&E staining of a section of a tonsil, containing a germinal centre and surrounding lymphocytes. b) p120-1 (6H11 mAb) and p120-3 (anti-p120-3) expression in a germinal centre and surrounding lymphoid tissue of the tonsil, and vimentin, PCK and E-cadherin in an adjacent section.

Supplementary figure 12. p120 isoform localization in the placenta and testis. Panels show p120-1 (6H11 mAb) and p120-3 (anti-p120-3) expression, and vimentin, PCK and E-cadherin and H&E in adjacent sections. For the placenta, the localization of a chorionic villus, the syncytiotrophoblast (SCTB) and a cytotrophoblast (CTB) are indicated.

Supplementary figure 13. Uncropped western blots from Figure 1 and Supplementary Figures 1 and 2.

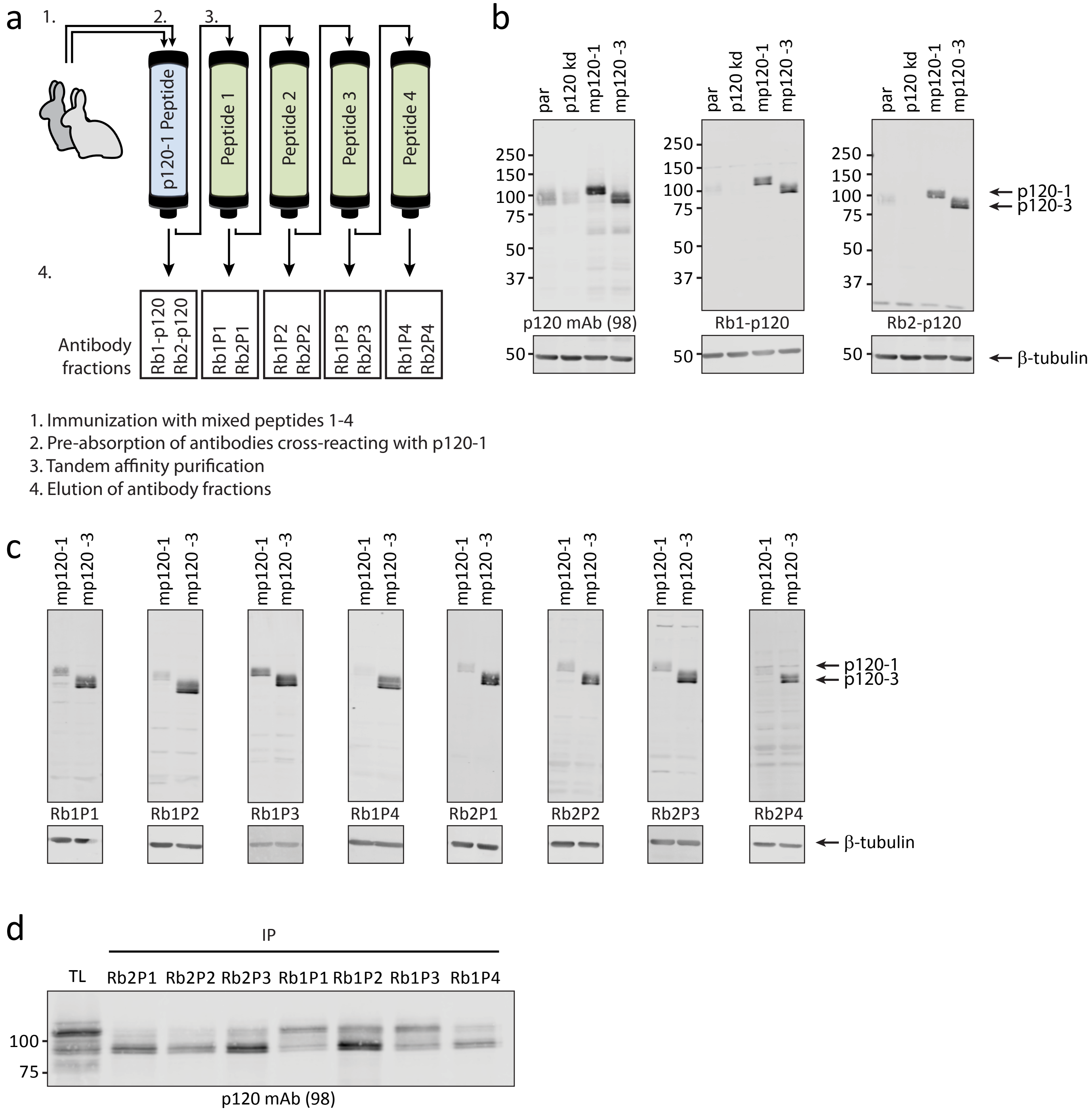

Supplementary Figure 1

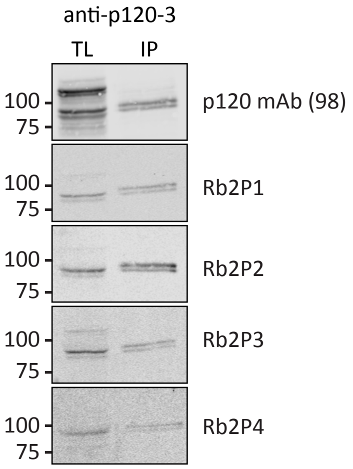

Supplementary Figure 2

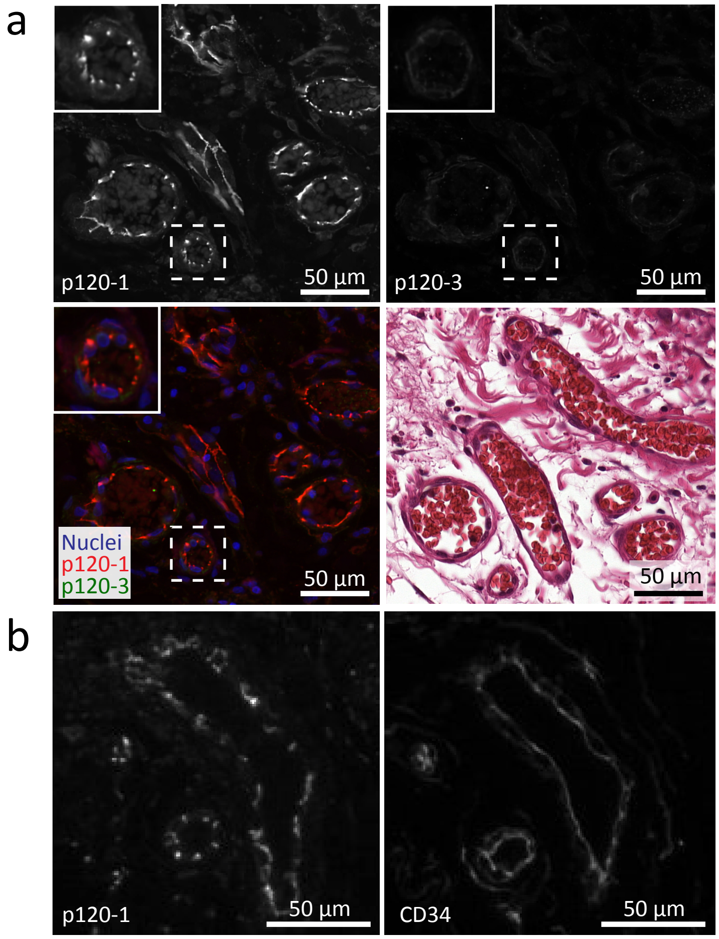

Supplementary Figure 3

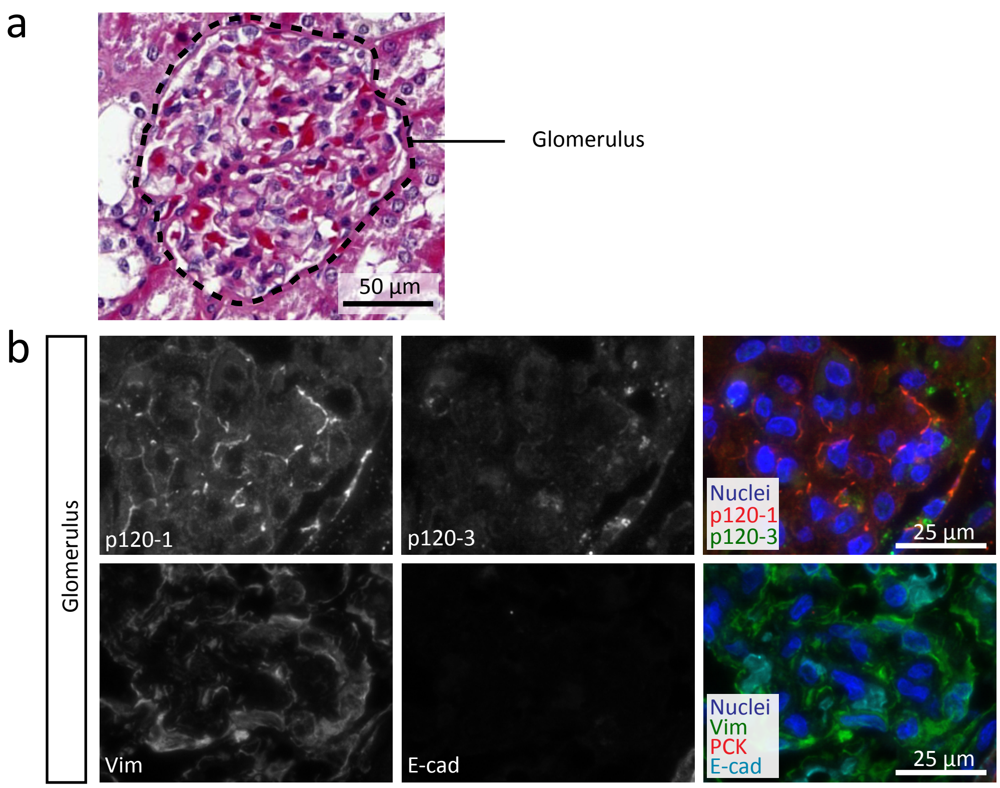

Supplementary Figure 4

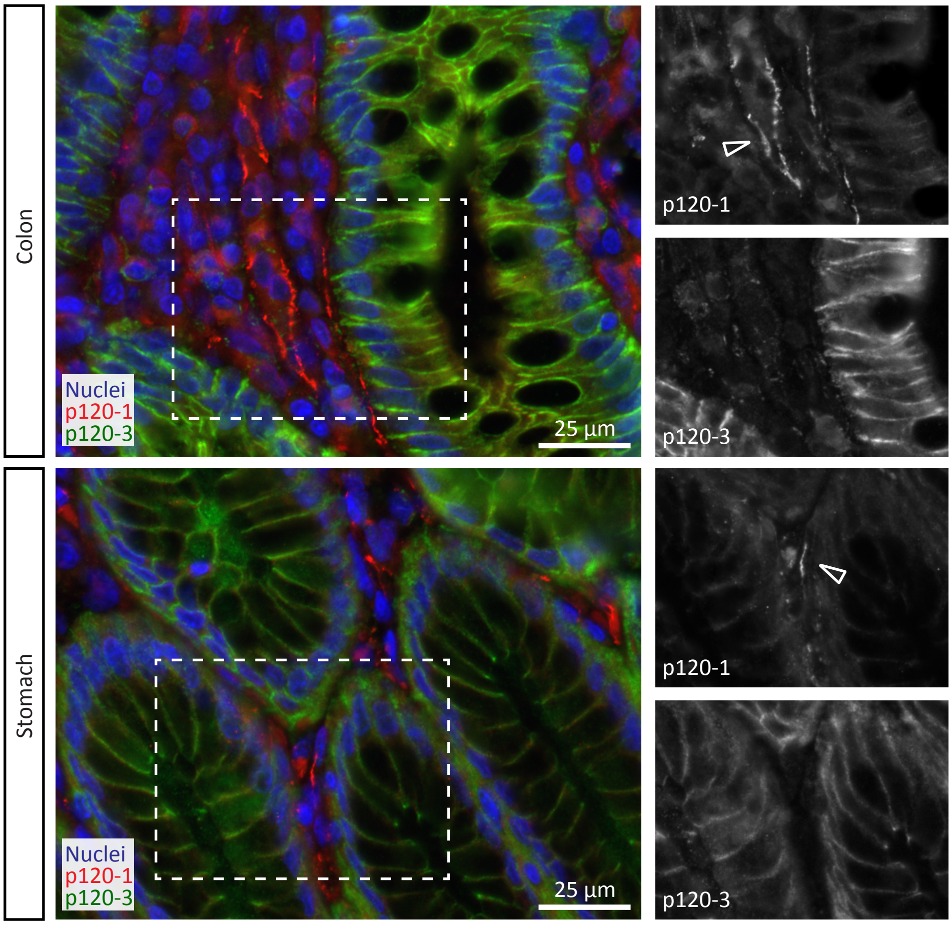

Supplementary Figure 5

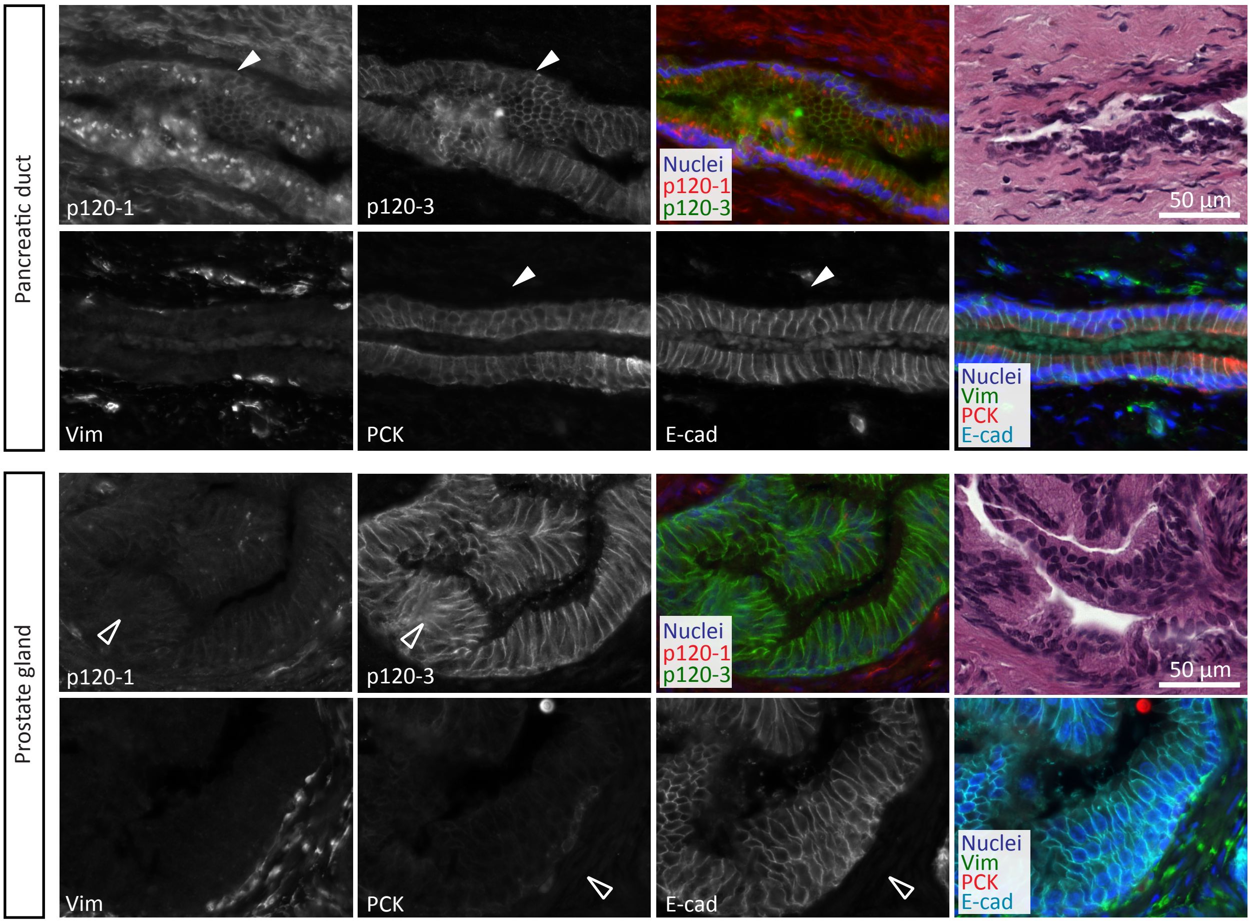

Supplementary Figure 6

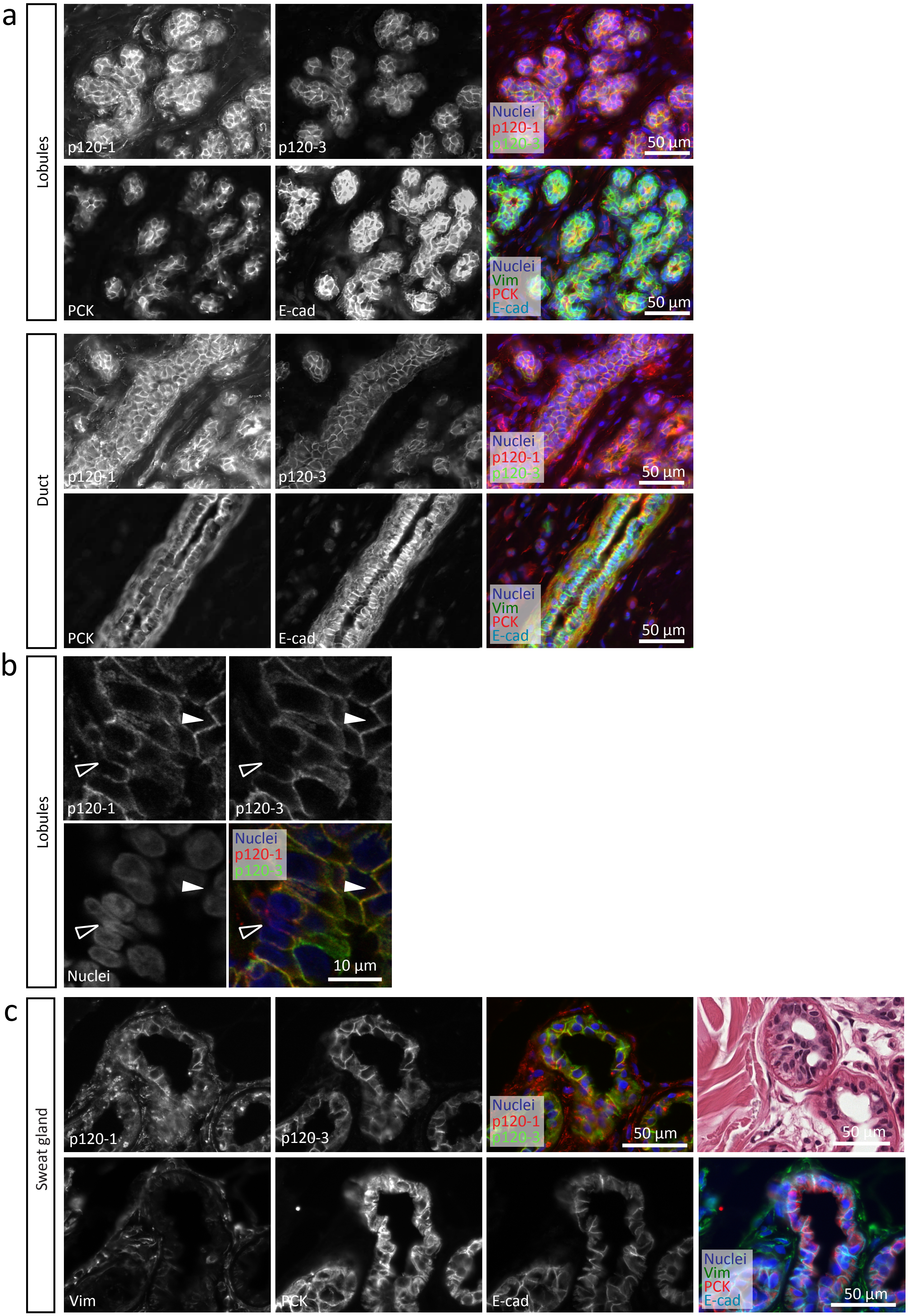

Supplementary Figure 7

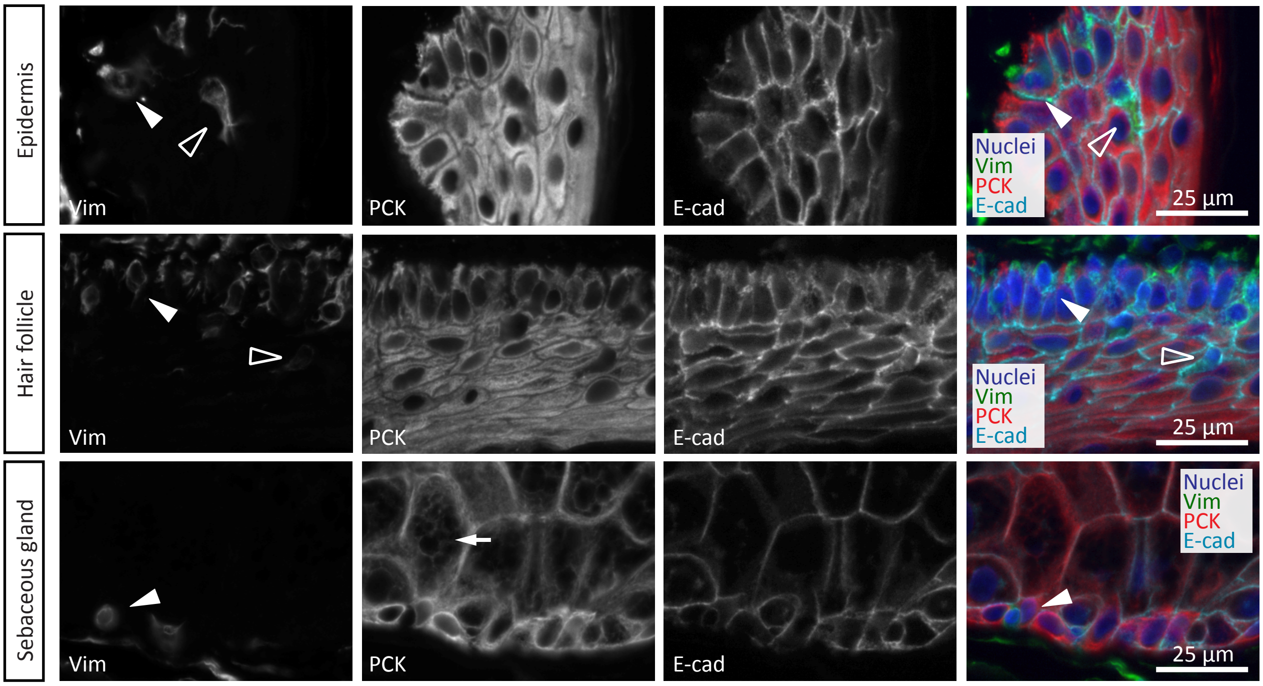

Supplementary Figure 8

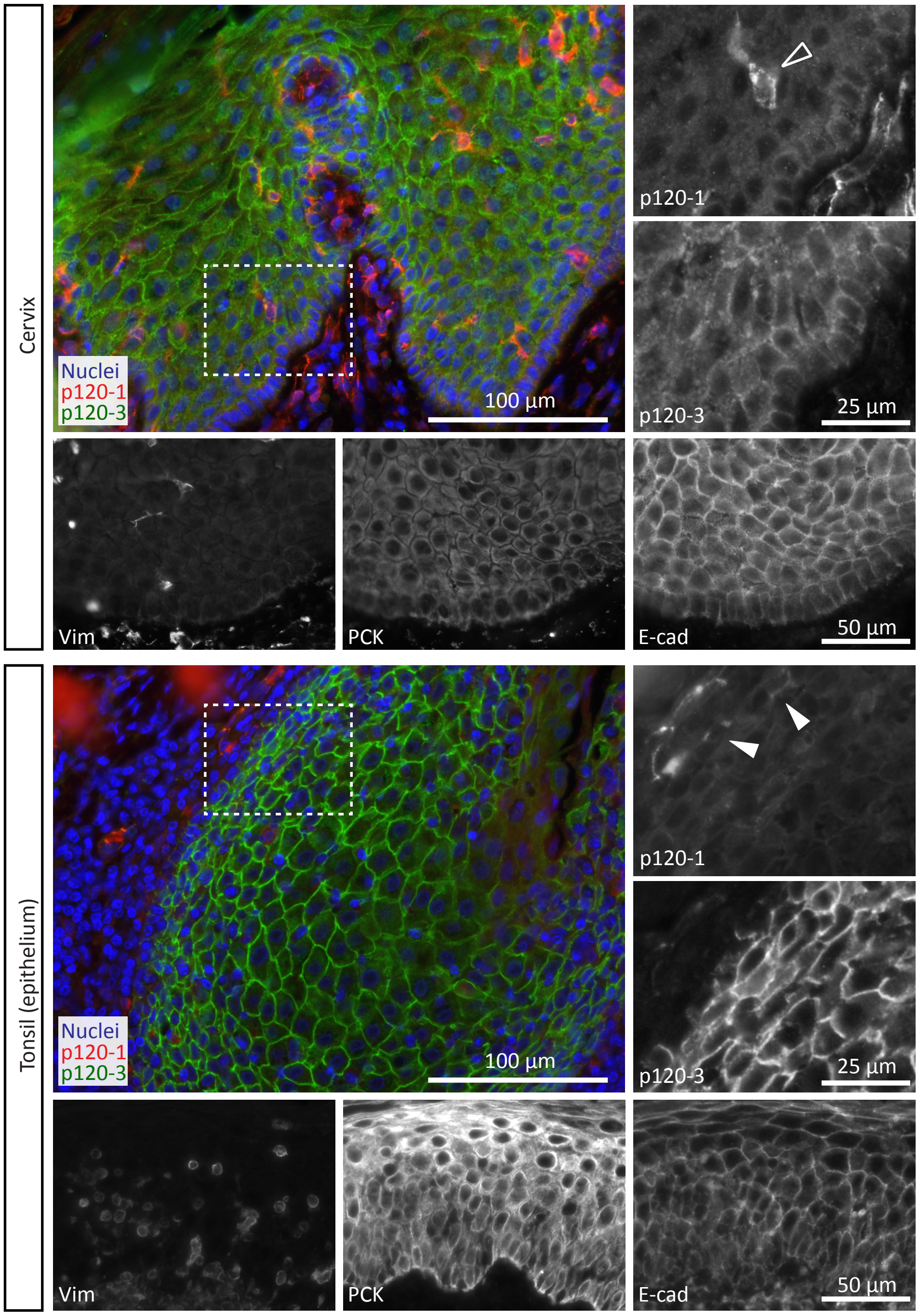

Supplementary Figure 9

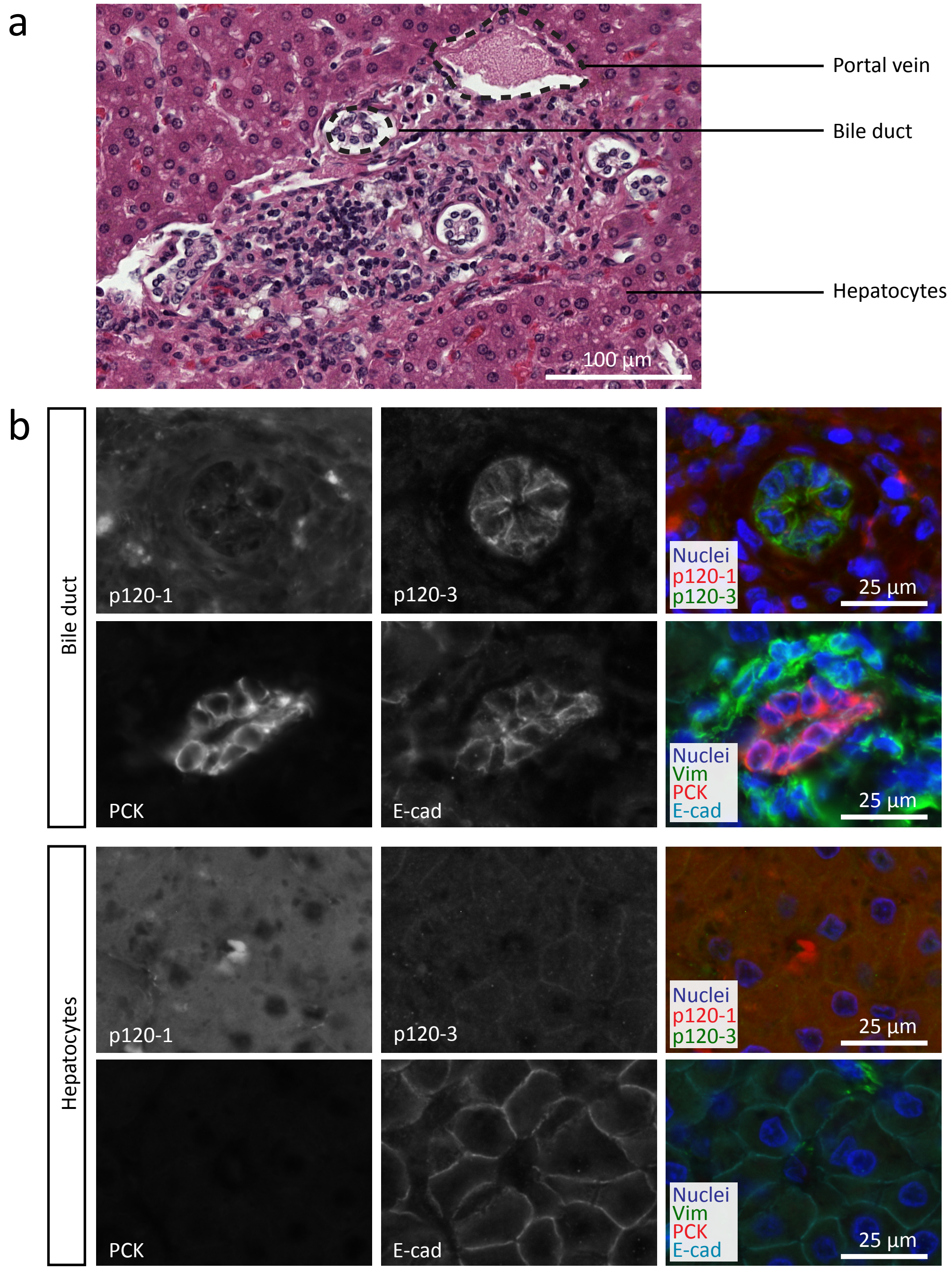

Supplementary Figure 10

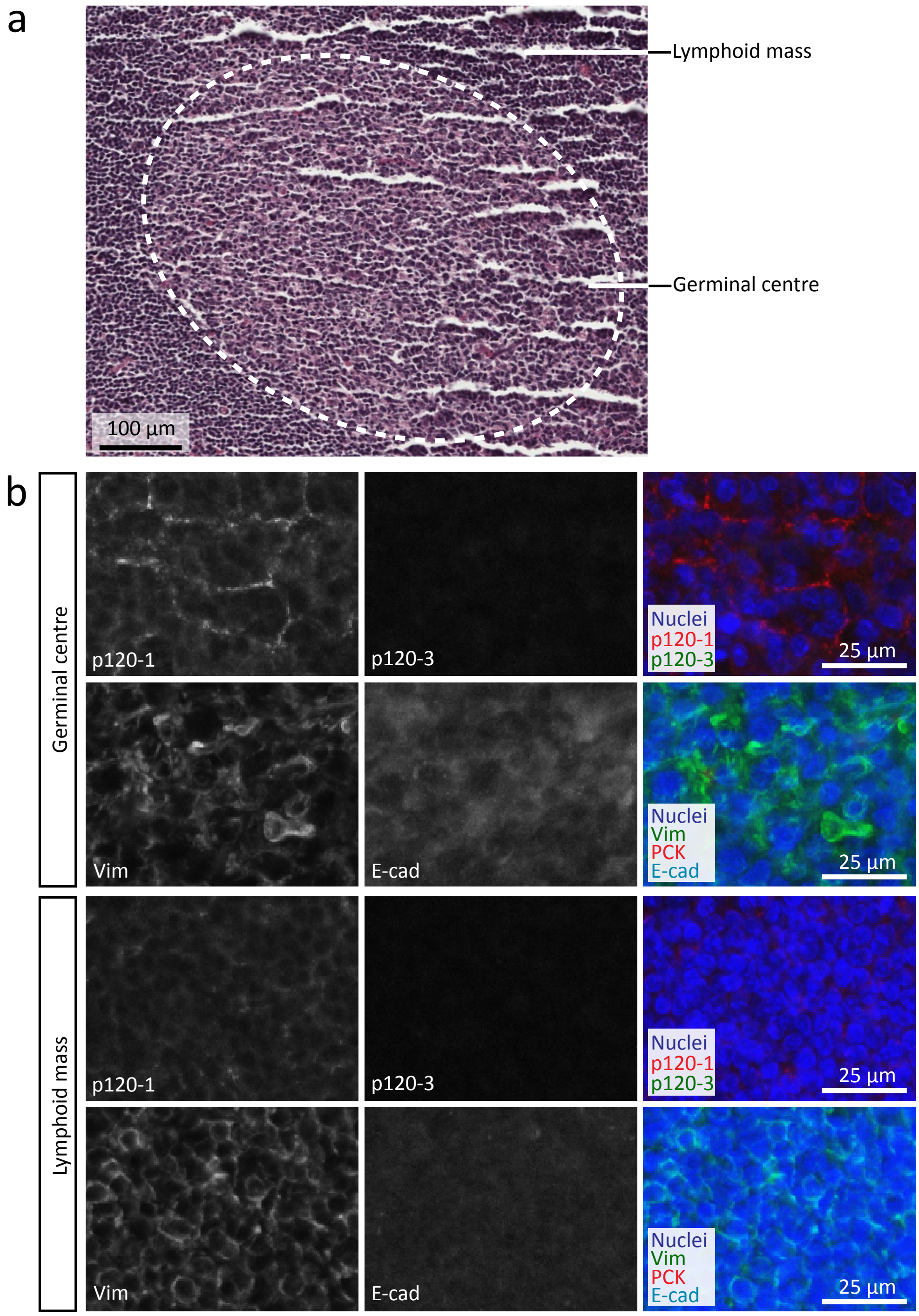

Supplementary Figure 11

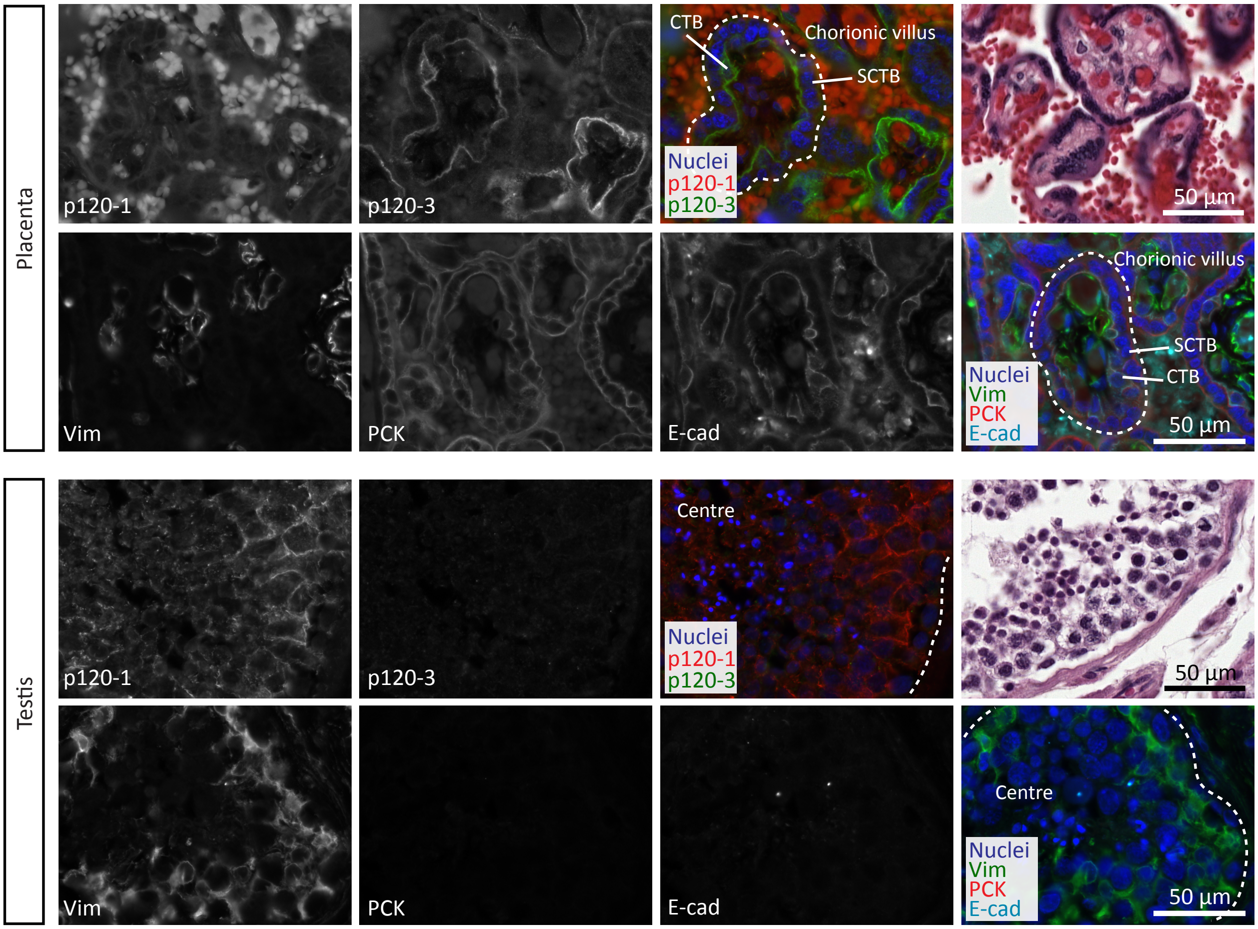

Supplementary Figure 12

Supplementary Fig. 1b

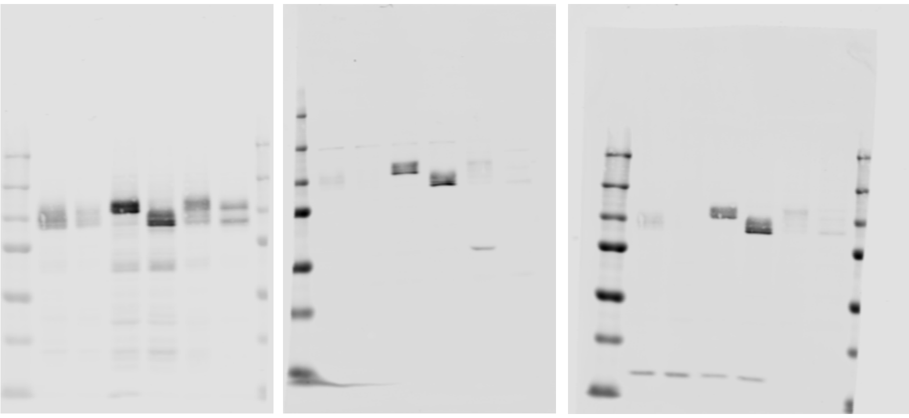

Supplementary Fig. 1d

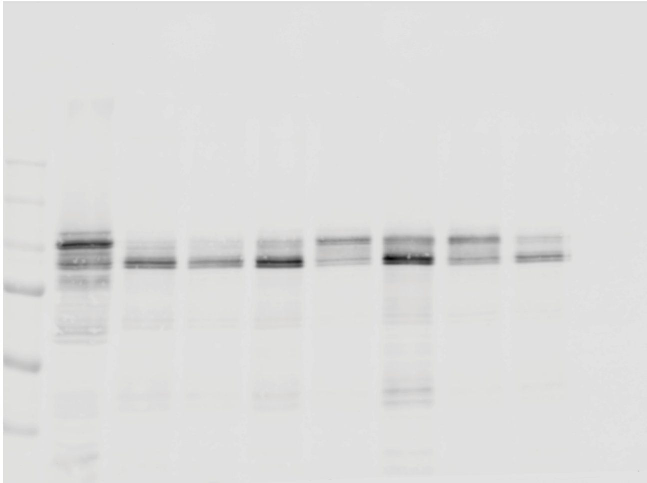

Supplementary Fig. 1c

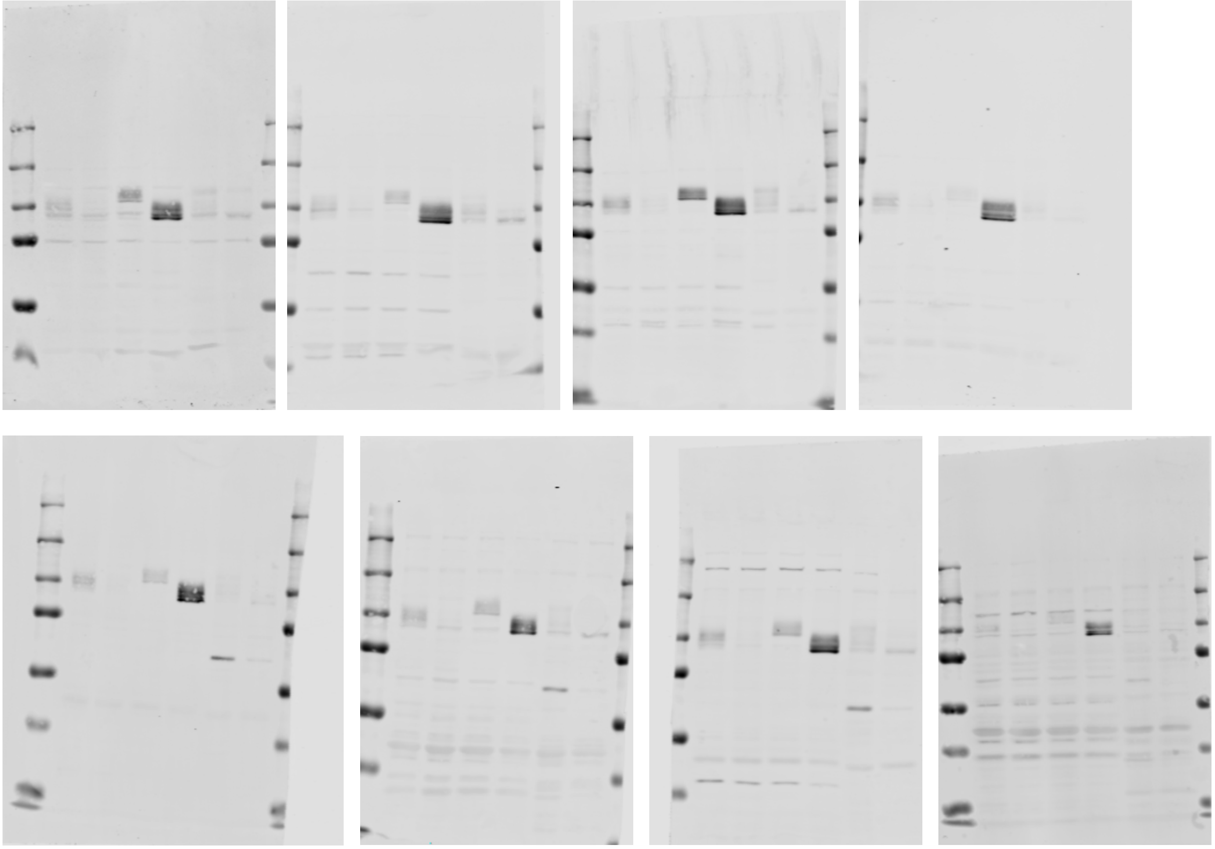

Fig. 1c

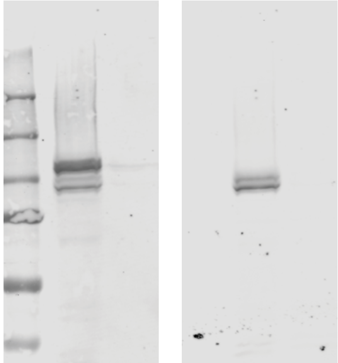

Supplementary Fig. 2

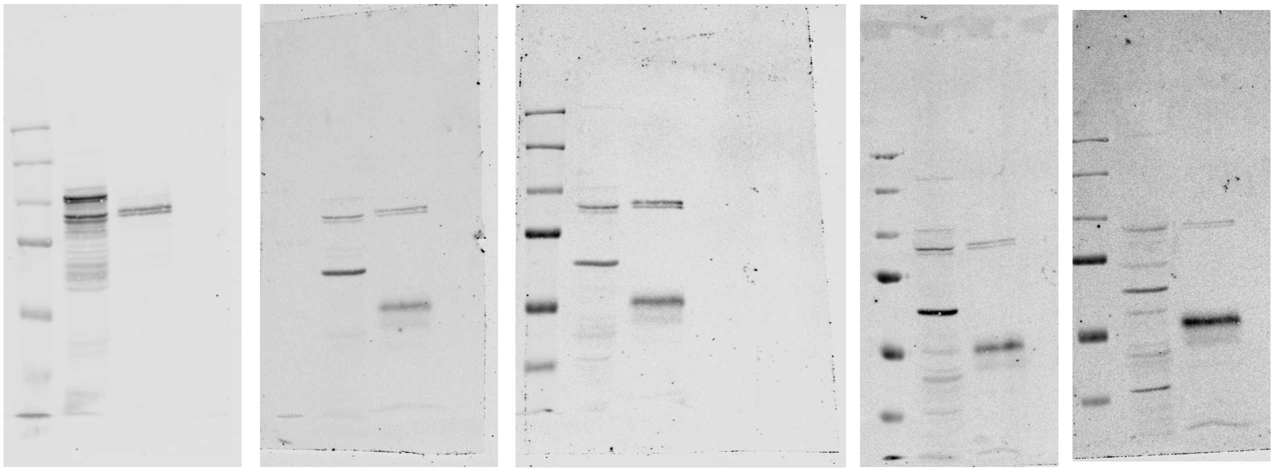

Supplementary Figure 13
